# “Not just a hobby, but a lifestyle”: Characteristics, preferences and self-perception of individuals with different levels of involvement in birdwatching

**DOI:** 10.1371/journal.pone.0255359

**Published:** 2021-07-30

**Authors:** Emilia Janeczko, Adrian Łukowski, Ernest Bielinis, Małgorzata Woźnicka, Krzysztof Janeczko, Natalia Korcz

**Affiliations:** 1 Department of Forest Utilization, Institute of Forest Sciences, Warsaw University of Life Sciences—SGGW, Warsaw, Poland; 2 Department of Silviculture, Faculty of Forestry, Poznan University of Life Sciences, Poznan, Poland; 3 Department of Forestry and Forest Ecology, Faculty of Agriculture and Forestry, University of Warmia and Mazury, Olsztyn, Poland; 4 Department of Forest Management Planning, Dendrometry and Forest Economics, Institute of Forest Sciences, Warsaw University of Life Sciences—SGGW, Warsaw, Poland; 5 Department of Natural Foundations of Forestry, Institute of Soil Science and Environment Management, University of Life Sciences in Lublin, Lublin, Poland; Shahjalal University of Science and Technology, BANGLADESH

## Abstract

Birdwatching is one of the most sustainable types of nature-based tourism and, at the same time, a form of recreation that is developing very dynamically. Birdwatching is attracting more and more people, not only professionals, but also amateurs from many countries. Birdwatching research is still relatively embryonic, especially when compared to nature tourism or wildlife tourism. Our main aim was to determine preferences and opinions of birdwatchers visiting the largest national park in Poland, in relation to their different levels of involvement. The data were collected in 2018 from a survey of a sample of 357 Polish and foreign birdwatchers. Results showed that birdwatcher respondents were predominantly male, middle-aged, and living in a large city. An important tool described in this article is a new scale that assesses the level of involvement of individual people engaged in birdwatching activity. This scale corresponds well with the individual characteristics of birdwatchers. Most birdwatchers defined their birdwatching activity as a permanent rather than a temporary hobby and therefore considered it to be more of a lifestyle than a hobby. Engagement in birdwatching activity increased with age and frequency of trips. The two most important reasons for birding were ‘to be close to nature’ and ‘fascination with birds’. It has been proven that the development of birdwatching in the future will require a developed infrastructure enabling interaction with the objects of observation.

## Introduction

There has recently been a growing interest among tourists in the natural environment, and demand for ecotourism increases annually [1–4]. Ecotourism is one of the fastest growing sectors in the tourism industry [3]. There are many benefits associated with nature tourism activities. For example, wildlife watching, wildlife photography, birdwatching, and birdfeeding are popular with many people and provides a significant economic contribution to local economies. Expenditures on wildlife-watching activities generate employment and income in various manufacturing industries and service sectors [5]. Thus in general tourists become more aware of the value of biodiversity and the conservation of natural resources [6], thereby the environmental and economic wellbeing of the local community improves [7, 8].

Avitourism, and therefore also birdwatching, is one of the most sustainable types of nature-based tourism [9, 10]. Birdwatching, defined as a nature tourism activity [11], is perceived as a form of recreation that provides an opportunity for contact with the natural world and an escape from modern, consumer-oriented society. However, Kronenberg [12] points out that wildlife viewing is nevertheless a visual experience and often requires evidence. By their presence and persistence, birdwatchers affect the attractiveness of birds’ breeding, migration or roosting sites; frighten birds; and otherwise increase pressure on birds and their habitats (e.g., by luring birds out of hiding places and stressing them by reproducing their calls, or by exposing birds and their nests to predators). Birdwatching is a relatively new activity for large groups of people that emerged at the beginning of the 20th century [12]. Previously, birdwatching was carried out by specialists, mainly ornithologists. Currently, these activities involve not only professionals, but also amateurs from many countries; birdwatching has developed most rapidly in the United States and western Europe, particularly in the UK, the Netherlands and Germany [13]. The study by Cordell and Herbert [14] shows that 70.4 million people in the United States are interested in birdwatching, indicating that birdwatching is one of the favourite activities of Americans. In Poland, only 7,000–10,000 people currently claim to engage in birdwatching, although estimates conducted by the Polish Society for the Protection of Birds show that we have about 3,000 birdwatchers in the country, which is a rather small number considering the area of the country and the total population [15].

Steven et al. [16] suggest that birdwatching research is still relatively embryonic and that research interest in avitourism is still much smaller compared to interest in nature tourism or wildlife tourism. The dominant issue in birdwatching research to date has been the recognition of the influence of demographic factors on birding behaviour [e.g. 9, 11, 16–22]. Subsequent studies have examined the environmental preferences of avitourists [13, 21, 23–25] The economic benefits of birdwatching development are also increasingly the subject of research [26–28].

There have also been several attempts to determine the extent to which people engage in this recreational activity. For example, Boxall and McFarlane [29] asked birders to rank themselves as casual, novice, intermediate or advanced, based on level of activity and intensity of interest. Scott and Thigpen [21] identified four groups of birders, namely casual, interested, active and skilled, but another study by Scott et al. [30] distinguished three measures of bird specialization: committed birders, active birders, casual birders. Vaske et al. [19] considered birdwatching within the broader category of wildlife viewing and distinguished four types of participants of recreational movement related to wildlife observation: highly involved tourists (highly engaged), creative tourists (creative), generalists (generalist) and casual tourists (occasionalist). Anderwald [31] distinguished five stages in the development of a birdwatcher’s knowledge: the silent keeper, the researcher-observer, the bird lover, the ornithological tourist and the Icarus man. In turn, Hvenegaard [23] divided birders into three distinct groups: advanced-experienced, advanced-active and novice. It is therefore clear that birdwatching is a form of recreation that encompasses various aspects of skills and knowledge, behaviour and stages of involvement that have a direct bearing on lifestyle. Thus, there is a lack of consistency in the nominal scales used to assess birdwatchers’ involvement in their hobby. Also it is noticeable that there are no proper guidelines to compare the results for any aspect of birdwatchers. Even the authors themselves change the scales they use, probably in search of better solutions [21, 30]. There is a need to find some simple measure that would define well the level of involvement in the activity of birdwatching. However, the best solution would be to use a different type of scale (other than a nominal scale) to harmonise this measure across studies.

Our main aim was to determine preferences and opinions of birdwatchers visiting the largest national park in Poland in relation to their different levels of involvement. An additional goal was to create a tool that would allow for a very precise expression of the level of involvement in birdwatching (using an interval scale) in order to address the lack of unification of the results obtained by different authors for groups of different levels of involvement. The significant rate of birdwatching development indicates the need for a better understanding of the demography, preferences and behavioural patterns of birdwatchers [6]. The results of preference studies may be useful in ensuring the multifunctional development of natural-valued areas [32]. Also Reichhart and Arnberger [33] believe that understanding visitor preferences is valuable in developing effective landscape management strategies. Preferences can help develop avitourism products that meet the needs of individual birdwatchers and help them plan more enjoyable experiences at their destinations [25]. In addition, Randler et al. [11] point out that knowledge of birdwatchers’ behaviour is important because it can have major implications for data collection and long-term analyses of bird data.

## Materials and methods

### Study area

This study was performed in Poland. Poland is located in central-eastern Europe and covers over 300,000 km^2^. The country’s population is close to 38 million people, giving an average population density of 120 people/km^2^. To practise birdwatching in Poland, it is not necessary to obtain a special license or course regarding knowledge. Birdwatchers are bound by common rules of behaviour in natural areas, as well as special restrictions in protective areas. It is also not necessary to be a member of any association. As individuals become involved in advanced work with birds (catching, ringing, measurements and scientific assistance, etc.), additional approvals and training courses may be required from them.

The Biebrza Valley is the most important breeding area for many species of wetland birds in Poland and one of the most important in central and western Europe. This importance increases as wetlands disappear from the European landscape. The Biebrza Valley belongs to the largest wildlife refuges in Europe and is of great importance for many species of feeding and resting birds during annual migrations. There are also boreal breeding species, as well as species whose geographic centre is in the taiga and tundra zone. The majority of this area is the Biebrza National Park, covered by the Ramsar Convention to protect the wetlands and bird breeding grounds. Birdwatching in the area is an opportunity to activate the local community. This is particularly important due to the fact that the area is located in the periphery, at a distance from larger cities, which means that living conditions here are not easy. Identification of needs, expectations and preferences of birdwatchers is necessary to create new tourist products and develop tourist services based on the principle of sustainable development.

### The questionnaire

Data were collected from a research survey prepared for the purposes of these studies. A sample of 357 Polish and foreign birdwatchers was taken in 2018. The participants are representative of the birdwatching population because they were selected from the entire spectrum of people visiting the Biebrza National Park. The survey was conducted in the field in the vicinity of infrastructure facilities dedicated to birdwatchers such as hides, observation towers and birdwatching terraces. Birders were defined, as in the Hvenegaard [23] study, as those visitors who visibly participated in birding activities (based on clues such as visiting popular birding sites and using binoculars, spotting scopes, and bird books). Participants were selected by scientists from Warsaw University of Life Sciences between April and October in 2018 in the park management building. We asked every fifth birdwatcher who visited Biebrza National Park in those days to complete the questionnaire by engaging in direct conversation with an interviewer who recorded the responses in paper form (n = 357). The questionnaire was prepared in several basic languages (Polish, German and English). Respondents were asked to provide information regarding their gender (male, female), age (18–34 years, 35–54 years, ≥55 years), level of education (primary, secondary, higher), place of living (village; ≤100,000 residents; >100,000 residents) and country of origin (native, abroad). Respondents provided information regarding their country of permanent residence, and we defined people of Polish nationality as native, because the research area belongs to the territory of Poland. They were questioned on many different topics that are presented and discussed in more detail in the Results and Discussion sections. Our research survey also allowed us to determine how birdwatchers’ preferences regarding the active realization of their hobbies in the field are formed. Respondents also had an opportunity to express an opinion on the subject of birdwatching in Poland. The time that respondents needed to complete the questionnaire ranged from 5 to 7 minutes.

The survey was conducted in full agreement with the national and international regulations in compliance with the Declaration of Helsinki (2000). The personal information and data of the participants were anonymous according to the General Data Protection Regulation of the European Parliament (GDPR 679/2016). The research was voluntary and did not take into account minors. In Poland, research of this type does not require the approval of the bioethical commission.

The questionnaire used in this study was fully anonymous. We did not collect any sensitive, personal information. Participation in the study was completely voluntary and informed. Respondents were not obligated in any way to participate in the study, they could refuse to participate and/or stop the interview at any time. Before the interview began, each respondent verbally expressed their willingness to participate in the study. We surveyed only adults. There is no question of an ethical violation in this situation. The consent of the university committee was not required in this regard.

### Data analysis

We wanted to find some simple measure that defines well the level of involvement in the activity of birdwatching. In this study, we developed an ‘involvement score’ based on six statements defining respondents’ reasons that prompted them to take up this hobby (motives) and on respondents’ involvement in six important activities related to birdwatching (performed activities; **Table 1**). The items that we used were selected on the basis of literature, our experience and observations of important issues related to birdwatching. We used principal component analysis to assess the construct validity of items and Cronbach’s α to measure the internal consistency [34]. To allow the use of Likert’s five-point scale, respondents were given the following five choices when they assessed their motives: definitely yes (+2), rather yes (+1), hard to say (0), rather no (–1), definitely no (–2). Thus, the score for motives ranged from –12 to +12 accounting for the responses to all six statements. To this, we added the results corresponding to respondents’ performed activities. It was a multiple answer question, where each selected option was awarded 1 point. There were 0 points awarded for not choosing a single answer. The combined, final involvement score for each respondent could range from –12 to +18.

**Table 1:** Birdwatchers’ responses to statements about their motives and activities performed for birdwatching.

		**Items**	**n**_**1**_	**% yes**	**n**_**2**_	**% no**	**n**_**3**_	**% hard to say**		**Factor loading PC1**		**Factor loading PC2**	
**What motivated you to practice birdwatching?**	1	I am simply interested in broadly understood nature	353	99	2	0.5	2	0.5		0.15		0.25	
	2	I am interested in and impressed by birds	346	97	10	2.5	1	0.5		0.45		0.24	
	3	**I want to impress other people through my hobby**	44	12	268	76	45	12		-0.45		0.70	
	4	**I consider birdwatching as a fad**	76	21	248	70	33	9		-0.57		0.67	
	5	I like to actively rest in nature	301	84	27	8	29	8		0.27		-0.30	
	6	I want to take unique photos	187	52	120	34	50	14		0.35		-0.21	
		Items	n_1_	% of the indicated answers				Factor loading PC1			Factor loading PC2		
**What activities related to birdwatching do you engage in?**	1	I feed the birds and observe their behaviour	252	71				0.12			0.21		
	2	I hang nesting boxes for birds	92	26				0.19			0.12		
	3	I belong to various associations and groups of people with similar ornithological interests	126	35				0.64			0.28		
	4	I improve my photography skills to take better photos	215	60				0.47			0.25		
	5	I read the popular articles and scientific articles in the field of ornithology	185	52				0.61			0.36		
	6	I create my own ornithological notes (I publish some of them)	157	28				0.70			0.21		

We used non-parametrical Wilcoxon / Kruskal-Wallis statistical tests (due to the lack of normal distribution–Shapiro-Wilk W Test; W = 0.9542; P < 0.0001) and post hoc nonparametric comparisons for each pair by Wilcoxon method (P < 0.05) to compare differences in the involvement score among categories of respondents, and we used omega square (*ω*^*2*^) to investigate effect size, with cut-off levels of *ω*^*2*^ > 0.01 for a small effect, *ω*^*2*^ > 0.06 for a medium effect and *ω*^*2*^ > 0.14 for a large effect, as is widely accepted [35]. Abbreviation ‘SE’ means standard error of the mean.

## Results

In total, we surveyed 357 respondents, and none were excluded, such that we used the responses of all respondents for further analyses.

### General characteristic of respondents

Among respondents, the majority were men (58%). The age of the respondents was analysed in groups, and most people were aged 35–54 years (42%). In general, the remaining age groups were represented similarly (18–34 years = 31% and ≥55 years = 27%). Approximately one third of respondents (37%) lived in places with more than 100,000 inhabitants (village = 31% and places with no more than 100,000 inhabitants = 32%). The majority of birdwatchers came from Poland (71%). The remaining respondents (29%) came from different countries, such as UK, Germany, Netherlands, Belgium, Spain, Czech Republic, Austria and Italy; in our analyses we consider them collectively as foreigners. Nearly all respondents had higher education. Only 14% and 2% had secondary and primary education, respectively.

### Involvement score

Nearly all birdwatchers claimed that they were simply interested in broadly understood nature (**Table 1**). Nearly all also declared that they were interested in and impressed by birds. The next two items were constructed in such a way as to make the respondents reflect more deeply on birdwatching (items 3 and 4 were reversed in order to eliminate automatism during the examination). In both cases, the vast majority (about three quarters) did not agree with the statement that their hobby is a temporary fad or that they wanted to impress others with their hobby. Over 80% of respondents indicated that birdwatching is a convenient activity for them, which helps them to relax and rest in nature. More than half of the respondents agreed it was important to take unique photos during active birdwatching, but one third did not support this view.

In the second part, where respondents were asked about which activities related to birdwatching they engage in, they could select as many answers as they wanted (therefore, for each question, 100% means the total number of respondents). The activity of feeding birds and observing their behaviour was characterized by the highest percentage of responses. Hanging nesting boxes for birds turned out to be the least popular activity among the respondents. More than half of the respondents read the popular and scientific articles in the field of ornithology, but only less than a quarter of them create their own ornithological notes or publish some of them. A relatively small group of respondents (slightly more than one third) feel the need to associate with people with similar interests and therefore belong to various associations and groups of people with similar ornithological interests. Sixty per cent of respondents indicated that photography and the activities related to it are an important part of birdwatching.

**Table 1** Responses of birdwatchers to six statements defining their motives and six performed activities related to birdwatching. Loadings (from principal component analysis) of each item on principal component one (PC1) and two (PC2) are also shown. Items in bold specifically investigated negative motives (points for answers were assigned in the opposite way than for the rest). In order to simplify the answers from questionnaires, we changed ‘definitely yes’ and ‘rather yes’ to YES and ‘rather no’ and ‘definitely no’ to NO. Data were collected in 2018 from a sample of 357 birdwatchers visiting Biebrza National Park.

**Fig 1:**
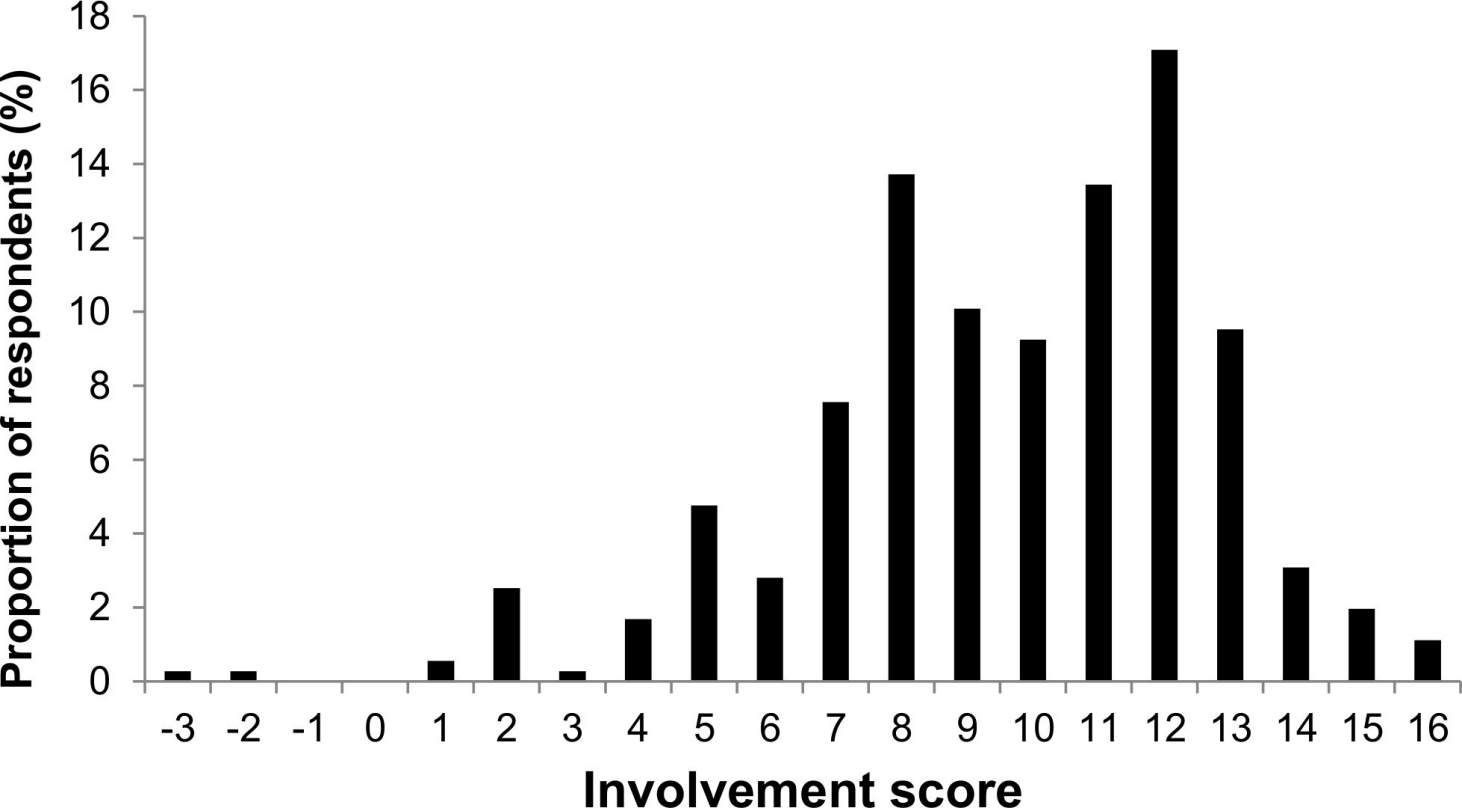
Percentage of birdwatchers with an engagement score.

The six statements defining respondents’ motives (Cronbach’s α = 0.52) and six important performed activities (Cronbach’s α = 0.57) had different factor loadings ranging from –0.57 to 0.69 on principal component 1 (PC1) and from –0.30 to 0.70 on principal component 2 (PC2), which were the only components with an eigenvalue >1 (PC1 1.84, PC2 1.25). PC1 explained 30.7% and PC2 20.8% of the variance, and thus we judged that it was sufficient to use these principal components only. The sum of these components was correlated with the involvement score (r = 0.694, P < 0.001), which is easier to interpret than PC1 or PC2. We therefore concluded that the involvement score is a suitable measure of involvement of birdwatchers in their activity. Almost all respondents (99.5%) held an involvement score that was at least slightly positive (i.e., had involvement score ≥1; **Fig 1**); mean involvement score was 9.66 (n = 357, SE = 3.1). This result indicates that the group of respondents includes only people who are actually, to a greater or lesser extent, associated with birdwatching.

**Fig 1** Histogram showing the proportion of birdwatchers with each involvement score. Involvement score was based on the six statements defining respondents’ motives and six important performed activities of birdwatching and ranged from –12 to +18 (Materials and Methods). Data were collected in 2018 from a sample of 357 birdwatchers visiting Biebrza National Park.

### Detailed characteristics, preferences and opinions of respondents

#### Characteristics

When analysing socio-demographic items, it should be stated that the group of birdwatchers was very homogeneous in terms of their involvement in their activity, as most of the differentiating features are statistically insignificant (**Table 2**). Only age turned out to be a differentiating factor with medium effect size, and the post hoc test showed that the involvement score increased with age.

**Table 2:** Average involvement scores of birdwatchers in relation to their socio-demographic characteristics.

Socio-demographic items (options)	Involvement score			P value	F	*ω*^*2*^	Effect size
	First option	Second option	Third option				
**Gender** (male, female)	9.61 ±0.22	9.69 ±0,24		0.8125	0.0564	0.00	-------
**Age** (18–34 years; 35–54 years; ≥55 years)	8.64c ±0.30	9.64b ±0.25	10.81a ±0.27	**<0.0001**	13.4301	0.07	medium
**Current place of residence** (village; ≤100,000 residents; >100,000 residents)	9.90 ±0.34	9.96 ±0.24	9.16 ±0.27	0.0726	2.6422	0.01	small
**Education** (primary, secondary, higher)	10.90 ±0.81	9.41 ±0.48	9.64 ±0.18	0.3820	0,9649	0.00	-------
**Country of origin** (native, abroad)	9.65 ±0.29	9.64 ±0.20		0.9731	0.0011	0.00	-------

**Fig 2:**
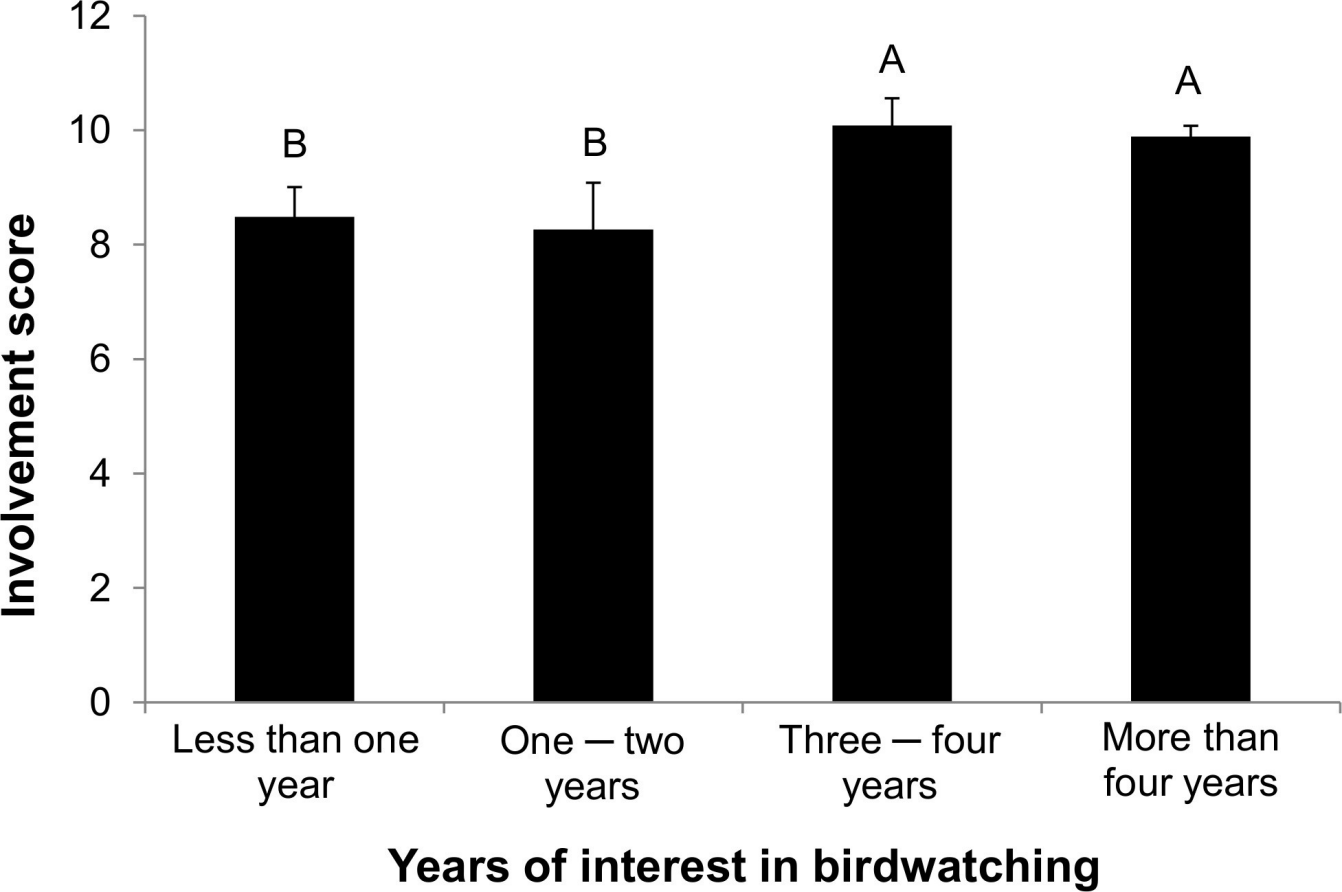
Mean involvement scores of birdwatchers in relation to time since their activity began (years of interest in birdwatching). Different letters indicate a statistically significant difference based on the post hoc nonparametric comparisons for each pair by Wilcoxon method (P < 0.05).

Involvement score were positively related with time since their activity began (years of interest in birdwatching), as personally assessed by each respondent (χ^2^_3,353_ = 9.10, P = 0.0280; **Fig 2**). People with more seniority (3 years and more) had higher scores than those with less seniority. It should be emphasized that the study group differed significantly in the case of *n*: the highest number of respondents declared that they were interested in birdwatching for more than 4 years (n = 260, 73%). Respondents who were interested in birdwatching for less than 2 years constituted only 17%.

The respondents were asked about their knowledge of specific species in the area where they intended to engage in birdwatching (Biebrza Valley). Each respondent was asked to provide four common or Latin names of birds. Involvement score were higher in people who properly reported the names of birds (9.93 ± 0.16 SE, χ^2^_2,354_ = 15.52, P < 0.0001) than in their counterparts who did not (7.50 ± 0.57 SE). It should be emphasized that the study group differed significantly in the case of *n*: the highest number of respondents properly reported the names (n = 317, 89%).

Involvement score was based on the six statements defining respondents’ motives and six important performed activities of birdwatching and ranged from –12 to +18 (Materials and Methods). Different letters indicate a statistically significant difference based on the post hoc nonparametric comparisons for each pair by Wilcoxon method (P < 0.05). Data were collected in 2018 from a sample of 357 birdwatchers visiting Biebrza National Park, Poland.

### Preferences

Half of the respondents declared that they participated in field trips to observe birds more than three times a year (n = 180). Slightly less than 30% of the respondents answered that they participated two to three times (n = 107), and the rest that they only participated once a year (n = 70). Involvement score results defining involvement of birdwatchers in their hobby were positively related with the frequency of field trips related to bird watching (χ^2^_2,354_ = 26.04, P < 0.0001). The highest number of points was obtained by the respondents from the groups who participated regularly (10.16 ± 0.23 SE) or at least two to three times a year (9.84 ± 0.26 SE), and these results were not statistically significantly different. People participating in birdwatching only once a year had 8.06 (± 0.37 SE) points and differed significantly from both above-mentioned groups.

Using a multiple-choice question, we checked what methods of observation were used by the respondents. About three-quarters (n = 270) of respondents used a variety of optical devices for observation. On the other hand, observations with no instruments were made by approximately 17% (n = 61). One-third of the respondents also used the method of listening to bird sounds (n = 119). Moreover, about 17% of the respondents (n = 62) used artificial shelters in their observations, in the form of shelters, tents or camouflage nets. Among those who had never used an additional shelter, around 15% declared that they were considering doing so in the near future.

**Fig 3:**
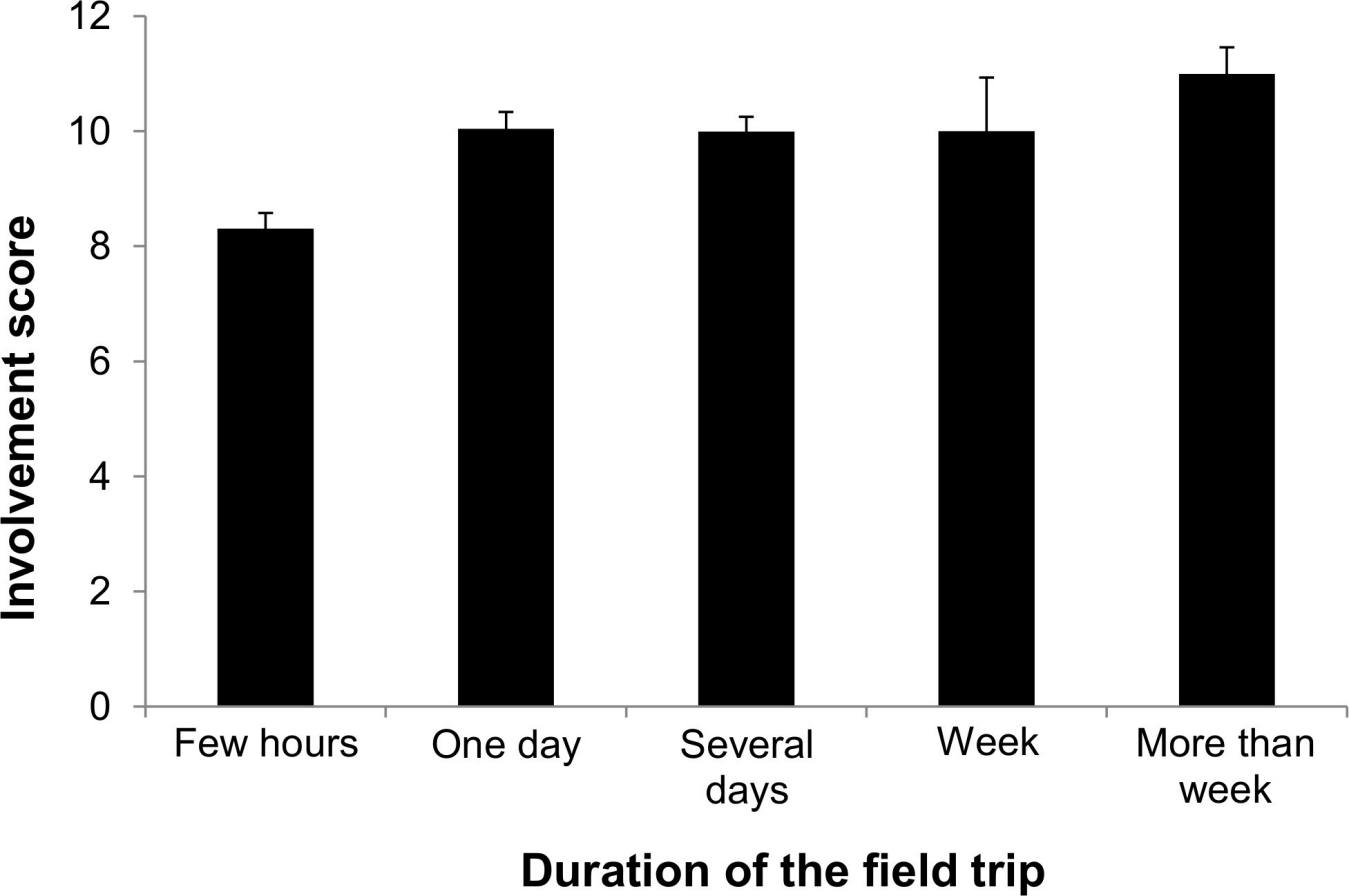
Mean involvement scores of birdwatchers in relation to duration of the field trips in which they participated.

Mean score results were positively related with duration of the birdwatcher’s field trips (**Fig 3**). People who took longer trips had much higher involvement scores. It should be emphasized that this question was multiple choice, so it is hard to specify whether the groups differed significantly. The highest number of respondents declared that they usually participated in trips lasting a few hours (n = 129). For the remaining respondents, we obtained the results n = 98, n = 110, n = 13, n = 40 for trips lasting 1 day, several days, 1 week and more than 1 week, respectively.

Respondents were linked to their favourite observing sites, as 91% said that they often went to the same sites to observe birds. They also declared that during birdwatching, they engaged in other forms of recreational activity, such as walking, fishing, etc. (n = 311, 87%).

### Opinions

As part of our research, we asked respondents a number of detailed questions to define their opinion on factors determining the development of birdwatching in Poland (**Table 3**). In general, it can be concluded that they think that the greatest influence on the development of birdwatching was due to factors such as natural values of Poland, social networks and general public interest in ecotourism. Among the factors assessed, they indicated that birdwatching infrastructure, prices of equipment helpful in observation and unsatisfactory offerings of travel agencies potentially inhibited the development of birdwatching in Poland.

**Table 3:** Responses of birdwatchers regarding selected factors determining the development of birdwatching in Poland and requirement of specialized infrastructure for birdwatching in Poland.

		Items	n_1_	% yes	n_2_	% no	n_3_	% hard to say
Do these selected factors determine the development of birdwatching in Poland?	1	Natural values of Poland	349	98	4	1	4	1
	2	Diverse and available for birdwatching infrastructure	173	48	96	27	88	25
	3	Affordable prices of equipment helpful in observation	153	43	95	27	110	31
	4	Wide range of offers from travel agencies	104	29	142	40	111	31
	5	Popularization of birdwatching in the media	215	60	92	26	50	14
	6	Wide offer of publications about birds	244	68	36	10	77	22
	7	Development of social networks enabling the exchange of experiences	290	81	26	7	41	11
	8	General public interest in ecotourism	267	77	40	11	40	11
Does the development of birdwatching in Poland require specialized infrastructure such as:	1	Observation tower	326	91	21	6	10	3
	2	Ground observation points	331	93	11	3	15	4
	3	Educational paths	294	82	36	10	27	8
	4	Platforms and viewing terraces	315	88	19	5	23	6

We asked two additional detailed questions. In the first question, we asked which specialized infrastructure was required in Poland to develop birdwatching (**Table 3**). The respondents indicated that basically each of the proposed options of infrastructure should help in development of their activity. Also, we asked about how often they used the currently available offerings of tourist offices. It turned out that most of the respondents (n = 305, 85%) did not use such offerings, and others used them sporadically (n = 47, 13%) or always (n = 5, 2%).

In order to simplify the answers from questionnaires, we changed in **Table 3** ‘definitely yes’ and ‘rather yes’ to YES and changed ‘rather no’ and ‘definitely no’ to NO. Data were collected in 2018 from a sample of 357 birdwatchers visiting Biebrza National Park.

## Discussion

### Methodological aspects

In the research we used a survey questionnaire. It is a very popular research tool, which can be used to investigate the expectations and preferences of tourists and visitors to valuable nature areas. The questionnaire was used in birdwatching studies conducted by, among others: Dwyer [17], Williams and La Montagne [18], Hvenegaard [23]. The questionnaire used in our study, after some modification, can be used to assess the degree of involvement in various forms of recreational activities based on nature (for example anglers, hunting). Due to the survey, we can predict how intensively birdwatching is developing, as well as the level of environmental awareness of its participants. The questionnaire we have constructed can also be used in the future to select people who can help in ornithological research (e.g. verification of knowledge of species, indication of breeding sites etc.). It can also be helpful for people preparing an individual offer for a tourist, adjusted to his/her abilities and level of involvement. The advantage of surveys is that they allow for relatively easy and quick testing, and that they guarantee the anonymity of respondents. However, it is not an ideal tool, it is very difficult to ensure the truthfulness of the data obtained. Therefore, we decided to use the form of interviews, in which questions were read out and the interviewer recorded the respondents’ answers. Direct contact makes it easier for the researcher to determine whether the answers obtained can be treated as reliable.

### General characteristics of respondents

In our study, birdwatchers were predominantly male, similar to those of Frątczak et al. [13] and Scott and Thigpen [21]. In the study of Ellis and Vogelsong [6], women were slightly over-represented, but, as the authors explained, this was probably due to the fact that in the male-female two-person groups encountered in the field, most often only one questionnaire was returned, and it was completed by a woman. Also, results from Adams et al. [36], Lee et al. [28] and Conradie [25] indicated that the proportion of women interested in birdwatching was higher than men. However, in both articles of Lee et al. [28] and Conradie [25] the respondents were not, as in our study, birdwatchers met in the field, but participants in festivals, large events organized with avitourism in mind. Certain demographic characteristics, mainly gender and age, in addition to socio-economic characteristics, are linked with cultural consumption patterns and participation in cultural events. Women, in general, are known to be more active consumers of cultural products than men [37]. Also, mature aged individuals were more represented among tourists particularly at some attractions such as cultural festivals [37]. A study by Kim et al. [38] found that women were 1.28 times more likely than men to attend local festivals and fairs. This might be the reason for the difference between the findings of Lee et al. [28] or Conradie [25] and our results in relation to the gender of birdwatchers. In our study, the proportion of older people, ≥55 years old, was approximately one-third of the total. Research by Dwyer [17] showed that participation in birdwatching increased with the age of observers. Birdwatching is mainly practiced by people aged 45–64 years. The respondents aged 18–24 years showed the least interest in birdwatching. This observation is confirmed by the studies of Ellis and Vogelsong [6] and Williams and La Montagne [18].

In our study, more than 80% of the respondents had a university degree. Also in the studies of Adams et al. [36] and Lee et al. [28], more than 70% of birders declared that they had a bachelor’s degree or higher.

In our study, approximately one-third of respondents each lived in towns, cities and villages. The results of Skłodowski and Jurkowska [39] indicated that Polish birdwatchers were predominantly city dwellers, especially in cities with more than 500,000 inhabitants. In turn, Dwyer’s [17] research indicated that among birdwatching participants, the most numerous group were residents of cities with populations below 50,000. In general, Dwyer [17] suggested that individuals living in areas with populations of less than 50,000 were more likely to participate in activities generally associated with substantial wildland areas than were individuals living in areas with larger populations. Activities requiring the development of specialized facilities or programs are more likely to involve individuals living in large urban areas, where there are usually substantial recreation facilities and programs. In Poland, due to socio-economic conditions, the tourism activity of rural residents is lower than that of urban residents [40]. Besides, as Seweryn and Niemczyk’s [41] study showed, rural residents preferred destinations with a different character than the environment in which they live every day (i.e., cities). Among the birdwatchers we surveyed, the largest group were Poles, but it is noteworthy that almost 30% of the respondents were residents of other European countries, mainly those (Great Britain, Germany, the Netherlands, Belgium) where birdwatching is developing very dynamically [13]. It could be stated that our respondent group is very similar to those described in previous reports from Poland, as well as to groups from other countries with a similar culture.

### Involvement score

In Adams’ [36] study, the two most important factors for birding were ‘to be close to nature’ and ‘fascination with birds’. These two factors also resonate in our research. Summing up first two items from **Table 1**, it can be said that the research group is quite homogeneous and is definitely aware of the relationship between birdwatching and nature. On the one hand, it is clear that birdwatching is a form of outdoor recreation activity, which, like fishing, backpacking, camping in a tent or hunting, for example, is strongly rooted in nature. On the other hand, it is also an activity that involves emotional, spiritual, physical and mental stimulation [25]. Fascination with birds leads to greater engagement, to acquiring more degrees of knowledge and to becoming a more professional ‘obsessive’ birdwatcher. It is therefore not surprising that the vast majority of respondents in our study disagreed with the statement that their hobby is a fad or that they wanted to impress others with their unique hobby. Birdwatching is a way of life and, at the same time, an activity that guarantees contact with nature and helps people to relax and unwind. The fascination with birds involves the search for ‘souvenirs’ or ‘trophies’ that prove that the visitor has actually visited a place or seen a particular species [12]. Hence, more than half of the respondents to our survey believe that it was important to take unique photos while actively birdwatching. Taking photographs is an important part of birdwatchers’ activities. This is also confirmed by the research of Skłodowski and Jurkowska [39]. Birdwatchers are people with high environmental awareness and nature sensitivity. They include both those who start their birdwatching by feeding birds (the highest response rate) and those who are characterized by a desire to obtain nature knowledge and also to share it with other people (just over one-third of respondents). More than half of the respondents in our study read popular scientific articles on ornithology, and approximately 25% of the respondents belonged to various associations and groups of people with similar ornithological interests.

One of the goals of our work was to address the lack of an appropriate tool to assess birdwatcher involvement in their activity. Researchers most often use various nominal scales or leave the assessment of involvement to the respondents. Usually, the use of such a scale prevents accurate comparisons of the results and some are indicative, and so we proposed a different approach. In this research, we used a different type of scale often used in other studies, an interval scale, to quantify involvement by assigning a point value to each respondent. It turned out that the values obtained, expressed by the involvement scores, corresponded well with the results of measurements of individual traits indicating involvement in birdwatching (e.g., increase of involvement scores of birdwatchers in relation to prolongation of duration of the field trips in which they participate). Therefore, we want to emphasize that the illustrated result in the form of a distribution close to normal (**Fig 1**) allows us to conclude that the set of issues used to express motives (six statements defining respondents’ motives) as well as activities performed (six important performed activities related to birdwatching) may be used in the future, during other studies. For example, similar tools have been created to unify the results achieved in the case of research on attitude toward hunting (attitude toward hunting score [42, 43]).

### Detailed characteristics, preferences and opinions of the respondents

Birdwatchers had a good knowledge of birds occurring in the area where they intend to practice their hobby (Biebrza Valley). They knew which bird species occur in this area, and they were determined to observe specific bird species, mainly *Acrocephalus paludicola* (Vieillot 1817), *Philomachus pugnax* (Linnaeus 1758), *Crex crex* (Linnaeus 1758), *Anser erythropus* (Linnaeus 1758) and *Clanga clanga* (Pallas 1811). Clearly, these were not casual tourists but people engaged in their hobby, devoting significant amount of time to it. It seems that Biebrza Valley met their expectations in this respect, as most of the respondents declared that they often went to the same places to observe birds. We also observed that involvement in birdwatching increased with age. This fact is confirmed by Frątczak et al. [13], who found that there were more older people among professional observers than among novice birdwatchers. This involvement increased with the length of the birdwatching period. The involvement of birdwatchers in their hobby was related to the frequency of field trips to observe birds. The majority of birdwatchers participated in nature observation trips more than three times a year, with trips most often lasting several hours. Also, research by Skłodowski and Jurkowska [39] indicated a prevalence of expeditions lasting mostly a few hours. With many hours of interaction with nature and focus, many experience a heightened sense of awareness and a higher level of interaction with the natural world. During expeditions, birdwatchers used, as both our studies have shown, many types of equipment, mostly optical devices. Research by Skłodowski and Jurkowska [39] found that the amount of equipment used increased with the level of professionalism of the birding participant. Birding is a broad concept, involving other senses, such as hearing, in addition to sight [15]. Hence, over 30% of our respondents also used the method of listening to bird sounds when birdwatching. The increased interest in birdwatching, or wildlife watching more broadly, leads to the development of a specialized leisure industry. There are situations when being invisible is the only way to observe rare and valuable species up close. Hence, the number of birdwatchers interested in using artificial shelters, tents or camouflage nets in their observations is also increasing, as shown, among others, by our research. The majority of our respondents participated in other activities when on a birding trip, such as walking or fishing. Also, a study by Conradie [25] showed that apart from engaging in birdwatching, birding tourism participants also pursued other activities such as observing wildlife, other animals, trees, wild flowers and butterflies. Interestingly, water-related recreational activities such as diving, snorkelling, beachcombing and boating were not as important.

Our research showed that the development of birdwatching corresponded with natural values. Research by Steven et al. [44] showed that birders were attracted to sites that provide high levels of biodiversity and presence of endemic species. With birdwatchers in mind, special facilities are being developed in the field (e.g., paths with points/chats for observing and photographing from hidden places, viewing platforms). This infrastructure makes it possible to multiply experiences, aesthetic sensations and provides unforgettable impressions [15]. Our research showed that respondents considered such facilities to be helpful in the development of the hobby of birdwatching, pointing above all to viewing towers and ground observation posts as the most desirable type of infrastructure. Ellis and Vogelsong [6] suggested that recreational infrastructure such as well-marked, accessible trails; quality signage; observation towers; and observational blinds could increase birders’ satisfaction. This is supported by Conradie [25], whose research showed that the most important attributes at birding destinations were accessible walking trails, information about birds, bird lists and possibility of spending time in bird hides.

Our research showed that the vast majority of birdwatchers did not use the offerings of tourist offices. Also Conradie [25] suggested that avitourists prefer to travel in pairs, small groups or independently. This means that birdwatching fits into the general trends of tourism development in Poland, set out in the government document “Tourism Development Programme to 2020” [45], accompanied by a change in tourists’ expectations. Visitors increasingly expect services tailored to their individual needs, providing a more authentic experience.

## Conclusion

An important tool described in this article is the new scale that allows assessment of the level of involvement of individual people engaged in birdwatching. This scale corresponded well with the individual characteristics of birdwatchers. Its statements were created based on the experience of the authors and literature. Statistically, the most common birdwatcher was male, middle-aged, living in a large city. Most birdwatchers defined their birdwatching activity as a permanent rather than a temporary hobby, and it was therefore considered to be more of a lifestyle. An important fact about the birdwatchers’ community is that people’s involvement in this activity increased with their age. It was also observed that the level of involvement in birdwatching activity was related to the frequency of trips. Birdwatchers also used equipment when observing birds. It has also been proven that certain natural values were related to the development of birdwatching in the opinion of the respondents and that the development of birdwatching in the future will require a developed infrastructure enabling interaction with the objects of observation. These insights are important for the future development of birdwatching tourism and can help guide the development of regional development strategies in the future.

## Supporting information

S1 File Survey questionnaire. (DOCX)
