# Supplementary material for: “Not just a hobby, but a lifestyle”: Characteristics, preferences and self-perception of individuals with different levels of involvement in birdwatching
Source: PLoS One. 2021 Jul 30;16(7):e0255359. doi: 10.1371/journal.pone.0255359 (PMC8323953; doi:10.1371/journal.pone.0255359)
Supplement: S1 File — (DOCX) [file pone.0255359.s001.docx]

**Supporting Information:** survey questionnaire in three languages

**BIRDWATCHING - AS A FORM OF TOURISM AND RECREATION**

Dear Sirs,

Birdwatching is an increasingly intensifying form of tourism and recreation in Poland. We would like to determine the profile of people interested in birdwatching and gather information about their tourist and recreational preferences. The collected data will be used to prepare scientific publications carried out jointly by the scienties of the Forestry Faculty of the Warsaw University of Life Sciences SGGW and the Biebrza National Park.

You are one of the people invited to participate in this study. The invitation was directed to people interested in developing birdwatching. Your opinions and answers indicated in our questionnaire are secured, confidential and anonymous.

**General information**

1. **Sex**
   - women
   - men
2. **Age**
   - 18-34 years
   - 35-54 years
   - over 55 years
3. **Place of resident**
   - village
   - city to 100,000 inhabitants
   - city over to 100,000 inhabitants
4. **Country of origin:……………………………………………………**
5. **Level of education**
   - primary
   - secondary
   - higher

**Detailed questions**

1. **Do these selected factors determine the development of birdwatching in Poland?**
   *please provide one answer in each row (answer on a scale from* definitely yes to definitely no)

- natural values of Poland
- diverse and available for birdwatching infrastructure
- affordable prices of equipment helpful in observation
- wide range of offers from travel agencies
- popularization of birdwatching in the media
- wide offer of publications about birds
- development of social networking sites enabling the exchange of experiences
- general public interest in ecotourism

1. **Does the development of birdwatching in Poland require specialized infrastructure such as:**

*please provide one answer in each row (answer on a scale from* definitely yes to definitely no)

- Observation towers
- Ground observation points
- educational paths
- platforms and view terraces

1. **How often do you participate in trips related to bird watching (on average in the year)?**

- once a year
- 2-3 times a year
- over 3 times a year

1. **Do you ever go to the same places to observe birds?**

- yes
- no

**5. How long is your time spent on birdwatching?**

- up to several hours
- whole day
- a few days
- week
- over week

**6. Do you use the offers of travel agencies or an professional guide / ornithologist to observe birds?**

|  | yes always | yes, sometimes | never |
| --- | --- | --- | --- |
| I use the offer of a travel agency |  |  |  |
| I use of the professional quide/ornithologist |  |  |  |

**7. What method of bird observation do you use most often?**

- listening to the sounds of birds
- observation without optical instruments
- obserwation with using optical instruments

**8. Do you do only birdwatching activities or over recreational things (like for example: fishing, cycling, walking)?**

- yes
- no

**9. How long have you been interested in birdwatching?**

not longer than one year

- 1-2 years
- 3-4 years
- over 4 years

**10. What activities related to birdwatching do you engage in??**

*You can chose several answers*

- I feed the birds and observe their behavior
- I hang nesting boxes for birds
- I belong to various associations and groups of people with similar ornithological interests
- I improve my photography skills to take better photos
- I read the popular articles and scientific articles in the field of ornithology
- I create my own ornithological notes (I publish some of them)

**11. What motivated you to practice birdwatching?**

*please provide one answer in each row (answer on a scale from* definitely yes to definitely no)

- I’m simply interested in broadly understood nature
- I'm interested in and impressed by birds
- I want to impress other people through my hobby
- I consider birdwatching as a fad
- I like to actively rest in nature

**12. Do you use special equipment during birdwatching (tents, shelters, camouflage nets)?**

- yes, I always equipped with this
- yes, I use this tape of this equipment when I take a photo
- no, but I think about this
- never

**13. Which kind of birds specific to the Biebrza Valley would you like to see first of all (please specify four species)?**

…………………………………………………………………………..

**Thank you very much**

**BIRDWATCHING - FORMA TURYSTYKI I REKREACJI**

Szanowni Państwo,

Birdwatching jest coraz intensywniej rozwijającą się formą turystyki i rekreacji w Polsce.
Naszym celem jest ustalenie profilu osób interesujących się birdwatchingiem oraz poznanie ich preferencji turystyczno- rekreacyjnych. Zebrane dzięki ankiecie dane posłużą do przygotowania publikacji naukowych realizowanych wspólnie przez pracowników Wydziału Leśnego SGGW w Warszawie oraz Biebrzańskiego Parku Narodowego.

Jest Pan/i jednym/jedną z uczestników/uczestniczek zaproszonych do udziału w badaniu. Zaproszenie zostało skierowane do osób zainteresowanych rozwijaniem birdwatchingu w Polsce. Poniższa ankieta jest anonimowa. Wskazane przez Państwa odpowiedzi są zabezpieczone, poufne i anonimowe.

**METRYCZKA**

1. **Płeć**

- kobieta
- mężczyzna

1. **Wiek**

- 18-34 lata
- 35-54 lata
- powyżej 55 lat

1. **Miejsce zamieszkania**

- wieś/osada
- miasto do 100 tys. mieszkańców
- miasto powyżej 100 tys. mieszkańców

1. **Województwo, na terenie którego Pan/i obecnie mieszka: ………………………………………………………..**
2. **Wykształcenie**

- podstawowe
- średnie
- wyższe

**Pytania zasadnicze**

- 1. **Jakie czynniki Pani/a zdaniem decydują o rozwoju birdwatchingu w Polsce?**
     *proszę udzielić jednej odpowiedzi w każdym wierszu (odpowiedź w skali od zdecydowanie tak do zdecydowanie nie)*
- walory przyrodnicze Polski
- bogata oferta sprzętu do obserwacji ptaków
- przystępna cena sprzętu do obserwacji ptaków
- szeroka oferta biur podróży
- popularyzacja birdwatchingu w mediach
- szeroka oferta publikacji poświęconych ptakom
- rozwój portali społecznościowych umożliwiających wymianę doświadczeń
- moda na ekoturystykę
  1. **Czy Pana/i zdaniem rozwój birdwachingu wymaga specjalistycznej infrastruktury rekreacyjnej takiej jak:**

*proszę udzielić jednej odpowiedzi w każdym wierszu (odpowiedź w skali od zdecydowanie tak do zdecydowanie nie)*

- wieże widokowe
- punkty obserwacyjne
- czatownie
- ścieżki edukacyjne
- pomosty i tarasy widokowe
  1. **Jak często w roku uczestniczy Pan/i średnio w wyjazdach związanych z obserwacją ptaków?**
- raz w roku
- dwa - trzy razy w roku
- powyżej trzech razy w roku
  1. **Czy zdarza się Panu/i wyjeżdżać w te same miejsca aby obserwować ptaki?**
- tak
- nie
  1. **Jak długo trwają Pani/a wyjazdy związane z birdwatchingiem?**
- do kilku godzin
- dzień
- kilka dni
- tydzień
- ponad tydzień

1. **Czy korzysta Pan/i z ofert biur podróży lub doświadczonego przewodnika/ornitologa wyjeżdżając w celu prowadzenia obserwacji ptaków?**

|  | tak, zawsze | tak, sporadycznie | nie |
| --- | --- | --- | --- |
| korzystam z oferty biura podróży |  |  |  |
| korzystam z doświadczonego przewodnika |  |  |  |

1. **Jaką metodę obserwacji ptaków stosuje Pan/i najczęściej?**

- słuchanie odgłosów ptaków
- obserwacja bez przyrządów optycznych
- obserwacja za pomocą przyrządów optycznych

1. **Czy przy okazji birdwatchingu realizuje Pan/i też inne formy aktywności rekreacyjnej (np. wędkowanie, spacery itp.)?**

- tak
- nie

1. **Jak długo interesuje się Pan/i birdwatchingiem?**

- nie dłużej niż rok
- 1 - 2 lata
- 3 - 4 lata
- powyżej 4 lat

1. **W jaki sposób pogłębia Pan/i swoją wiedzę o ptakach?**

*można udzielić kilku odpowiedzi*

- dokarmiam ptaki i obserwuję ich zachowanie
- zakładam budki lęgowe dla ptaków
- przynależę do różnych stowarzyszeń i grup ludzi o podobnych-ornitologicznych zainteresowaniach
- nie rozstaję się z lornetką/aparatem fotograficznym
- czytam branżową prasę, śledzę artykuły naukowe dotyczące ptaków
- tworzę własne notatki ornitologiczne
- inny sposób

1. **Co skłoniło Pana/ią do uprawiania birdwatchingu?**

*proszę udzielić jednej odpowiedzi w każdym wierszu (odpowiedź w skali od zdecydowanie tak do zdecydowanie nie)*

- po prostu interesuję się przyrodą
- interesują mnie ptaki
- chęć zaimponowania innym
- moda
- aktywny wypoczynek na łonie przyrody
- chęć wykonania niepowtarzalnego zdjęcia

1. **Czy w trakcie obserwacji ptaków stosuje Pan/i tzw. sztuczne ukrycia (namioty, szałasy, siatki maskujące)?**

- tak, zawsze jestem na to przygotowany
- tak, tylko w celu fotografowania
- nie, ale rozważam
- zdecydownie nie

1. **Jakie ptaki charakterystyczne dla Doliny Biebrzy chciałby Pan/Pani zobaczyć przede wszystkim (proszę wymienić cztery gatunki)?………………………………………………………..**

**Serdecznie dziękujemy**

**BIRDWATCHING - EINE FORM VON TOURISMUS UND REKREATION**

Geehrte Damen und Herren,

Birdwatching ist eine sich immer stärker entwickelnde Form von Tourismus und Rekreation in Polen. Unser Ziel ist es, das Profil von Personen, die an der Vogelbeobachtung interessiert sind, zu bestimmen und deren touristische und freizeitbezogene Präferenzen kennenzulernen. Die durch diese Umfrage gesammelten Daten werden zur Vorbereitung der wissenschaftlichen Publikationen verwendet, die von den Mitarbeitern der Forstwissenschaftlichen Fakultät an der SGGW (Zentrale Schule der Ländlichen Betriebswirtschaft) in Warschau realisiert werden.

Sie sind einer der Teilnehmer, die zur Teilnahme an der Befragung eingeladen wurden. Die Einladung richtete sich an Personen, die sich für die Entwicklung der Vogelbeobachtung in Polen interessieren. Die folgende Umfrage ist anonym. Die von Ihnen angegebenen Antworten sind geschützt, vertraulich und anonym.

**IMPRESSUM**

1. **Geschlecht**

- weiblich
- männlich

1. **Alter**

- 18-34 Jahre
- 35-54 Jahre
- über 55 Jahre

1. **Wohnort**

- Dorf/Siedlung
- Stadt bis 100 000 Einwohner
- Stadt über 100 000 Einwohner

1. **Das Land, in dem Sie zur Zeit wohnen:** ………………………………………………………..
2. **Ausbildung**

- Sekundärstufe I
- Sekundärstufe II
- Hochschule/Fachhochschule

**Grundfragen:**

- 1. **Welche Faktoren entscheiden Ihrer Meinung nach über die Entwicklung der Vogelbeobachtung in Polen?**
     *kreuzen Sie bitte in jeder Zeile eine Antwort an (antwort auf einer Skala von stark ja bis stark nein)*
- naturbezogene Vorteile Polens
- großes Angebot an Austattung zur Vögelbeobachtung
- günstige Preise der Austattung zur Vögelbeobachtung
- reiches Angebot der Reisebüros
- Popularisierung der Vogelbeobachtung im Fernsehen und Radio
- eine breite Palette von Publikationen über Vögel
- Entwicklung von Social-Networking-Portalen, die den Erfahrungsaustausch ermöglichen
- Mode für Öko-Tourismus
  1. **Finden Sie, dass die Entwicklung von Birdwatching eine spezialisierte Infrastruktur erfordert, wie z.B.:**

*kreuzen Sie bitte in jeder Zeile eine Antwort an (antwort auf einer Skala von stark ja bis stark nein)*

- Aussichtturme
- Beobachtungspunkte
- Verstecke
- Bildungspfade
- Plattformen und Aussichtsterrassen
  1. **Wie oft nehmen Sie an Reisen teil, die mit der Vogelbeobachtung verbunden sind?**
- einmal im Jahr
- zwei-, dreimal im Jahr
- mehr als dreimal im Jahr
  1. **Kommt es vor, dass Sie die gleichen Orte besuchen, um Vögel zu beobachten?**
- Ja
- Nein
  1. **Wie lange dauern Ihre Vogelbeobachtungsreisen?**
- bis ein paar Stunden
- einen Tag
- ein paar Tage
- eine Woche
- länger als eine Woche

1. **Gebrauchen Sie Angebote von Reisebüros oder einem erhahrenen Führer/Ornithologen bei Ihren Vögelbeobachtungsreisen?**

|  |  | Ja, immer |  | manchmal |  | Nein |
| --- | --- | --- | --- | --- | --- | --- |
| ich nehme Angebote eines Reisebüros in Anspruch |  |  |  |  |  |  |
| ich greife auf einen erfahrenen Führer zurück |  |  |  |  |  |  |

1. **Welche Methode der Vogelbeobachtung wird von Ihnen am häufigsten angewendet?**

- Hören der Vögelstimmen
- Beobachting ohne optische Geräte
- Beobachting mit Hilfe der optischen Geräte

1. **Betreiben Sie bei der Vogelbeobachtung auch andere Aktivitäten der Freizeitgestaltung (z. B. Angeln, Wandern usw.)?**

- Ja
- Nein

1. **Seit wann interessieren Sie sich für die Vogelbeobachtung?**

nicht länger als ein Jahr

- 1-2 Jahre
- 3-4 Jahre
- länger als 4 Jahre

1. **Wie erweitern Sie Ihr Wissen über Vögel?**

*mehrere Antworten sind möglich*

- Ich füttere Vögel und beobachte deren Verhaltensweisen
- Ich baue Nistkästen für Vögel auf.
- Ich gehöre verschiedenen Vereinen und Gruppen von Menschen mit ähnlichen ornithologischen Interessen an
- Ich trenne mich nicht vom Fernglas/von der Camera.
- Ich lese Fachpresse, ich verfolge wissenschaftliche Artikel über Vögel
- Ich erstelle meine eigenen ornithologischen Notizen
- auf eine andere Art und Weise

1. **Was hat Sie dazu veranlasst, Vögel zu beobachten?**

*kreuzen Sie bitte in jeder Zeile eine Antwort an (antwort auf einer Skala von stark ja bis stark nein)*

- Ich bin einfach an der Natur interessiert
- Ich interressiere mich für Vögel
- Ich will andere Menschen beeindrucken
- es ist Mode
- aktive Erholung in der Natur
- Wunsch, einzigartiges Foto zu machen
- etwas Anderes

1. **Benutzen Sie während der Vogeleobachtung künstliche Verstecke (Zelte, Hütten, Tarnnetze)?**

- Ja, ich bin dazu immer vorbereitet
- Ja, nur zum Fotografieren
- Nein, aber ich erwäge es.
- Definitiv nein

1. **Welche für das Biebrza-Tal spezifischen Vogelarten würden Sie am liebsten sehen (bitte vier Arten angeben)?: …………………………..**.

**Herzlichen Dank!**
